# Genome‐wide DNA methylation analysis of Hashimoto's thyroiditis during pregnancy

**DOI:** 10.1002/2211-5463.13018

**Published:** 2020-11-11

**Authors:** Cai Wenqian, Wenlei Fan, Xijiang Hu

**Affiliations:** ^1^ Eugenic Genetics Laboratory Wuhan Children's Hospital (Wuhan Maternal and Child Healthcare Hospital) Tongji Medical College Huazhong University of Science & Technology Wuhan China; ^2^ Internal Medicine DEPT Wuhan Children's Hospital (Wuhan Maternal and Child Healthcare Hospital) Tongji Medical College Huazhong University of Science & Technology Wuhan China

**Keywords:** DNA methylation, genome‐wide methylation, Hashimoto's thyroiditis, pregnancy

## Abstract

Hashimoto's thyroiditis (HT) during pregnancy is usually accompanied by an elevation of thyroid‐stimulating hormone and a reduction of serum‐free thyroxine during gestation, which may lead to abortion, preterm delivery, and reduced intellectual function of the offspring. Epigenetic alterations may provide important insights into genetic–environmental interactions in HT. Here, we examined global DNA methylation patterns in patients with HT during pregnancy. DNA was extracted from 13 women with HT during pregnancy (HTDP) and eight healthy pregnant women as a control group. Genome‐wide methylation was detected with the use of an Illumina Human Methylation 850K Beadchip. A total of 652 differentially methylated positions (DMPs) and 27 differentially methylated regions (DMRs) were identified between the HTDP and control groups. GO analysis revealed that DMPs were significantly enriched in 540 GO terms, which included regulation of the differentiation of keratinocytes, T helper cell differentiation, and alpha‐beta T‐cell differentiation. Moreover, significant enrichment of KEGG pathways of the DMPs included mucin‐type O‐glycan biosynthesis, focal adhesion, and the insulin signaling pathway. The GO items associated with DMRs included muscle cell proliferation, response to biotic stimulus, anatomical structure formation involved in morphogenesis, and genes primarily involved in the FoxO signaling pathway. Finally, the DTNA gene was identified as the seed gene of functional epigenetic modules. In summary, the DNA methylation pattern of the HTDP group was distinct from that of the control group, and thus, changes in DNA methylation may influence the development of HT by regulation of the autoimmunity process.

## Graphical abstract

Epigenetic alterations may provide important insights into genetic–environmental interactions in Hashimoto's thyroiditis (HT). A total of 652 differentially methylated positions and 27 differentially methylated regions were identified between patients with Hashimoto's thyroiditis during pregnancy (HTDP) and healthy pregnant women. DTNA was identified as the seed gene of functional epigenetic modules. The DNA methylation pattern of HTDP was distinct from that of healthy pregnant women, and thus changes in DNA methylation may influence the development of HT via regulation of autoimmunity.

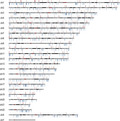

**Abbreviations**
anti‐Tg: anti‐thyroglobulin antibody
anti‐TPO: anti‐peroxidase antibody
CNV: copy number variation
CpG: cytosine–phosphate–guanosine
DMPs: differentially methylated position
DMRs: differentially methylated regions
DMSs: differentially methylated sites
GO: gene ontology
HT: Hashimoto's thyroiditis
HTDP: HT during pregnancy
KEGG: Kyoto Encyclopedia of Genes and Genomes
TAI: thyroid autoimmunity

The possible role of thyroid autoimmunity (TAI) in the context of infertility and pregnancy loss continues to receive increased attention. TAI is the most frequent underlying factor associated with thyroid function, affecting more than 17% of women of reproductive age [1, 2]. Hashimoto's thyroiditis (HT) during pregnancy is usually accompanied with an elevation of thyroid‐stimulating hormone and a reduction of serum‐free thyroxine during gestation, which could lead to abortion, preterm delivery, and reduced intellectual function of the offspring [3]. Moreover, maternal hypothyroidism during pregnancy is associated with significant pediatric morbidity due to infection [4]. Thus, a more effective strategy for the treatment of HT in pregnant women is urgently needed.

DNA methylation is a form of chemical modification during which methyl groups are added to the DNA molecule. Multiple cellular processes associated with DNA methylation have been reported, including genomic imprinting, repression of repetitive elements, and maintenance of cellular identity [5, 6]. Generally, methylation of cytosine–phosphate–guanosine (CpG) sites of the gene promoter is related to transcriptional inhibition, while DNA methylation of the gene body is positively correlated with gene expression [7, 8]. Many investigators have explored the potential role of DNA methylation in the development of hypothyroidism. For example, Wu *et al*. [9] reported that DNA methylation of the EphA5 promoter significantly influences the secretion of thyroid hormones. In the developing rat hippocampus, Li *et al*. demonstrated that DNA methylation might be related to the down‐regulation of the RELN and BDNF genes induced by hypothyroidism [10]. Meanwhile, a recent study found that DNA methylation was involved in the inhibition of thyroid gene expression [11]. However, the role of DNA methylation in the development of hypothyroidism during pregnancy remains unclear.

Thus, in order to explore the role of DNA methylation in the development of HT during pregnancy, DNA was extracted from 13 women with HT during pregnancy (HTDP) and 8 healthy pregnant women as a control group. Genome‐wide methylation was detected with the use of a DNA methylation microarray for 850 000 CpG sites of the human genome and the differences in DNA methylation between the two groups were further analyzed. Functional analysis was performed to investigate the potential biological functions associated with differentially methylated positions (DMPs) and differentially methylated regions (DMRs). In addition, functional epigenetic modules and copy number variation (CNV) were identified.

## Methods

### Subjects and methods study approval

This study protocol was approved by the Ethics Committee of Wuhan Children's Hospital and conducted in accordance with the ethics standards regarding human described in the Declaration of Helsinki (2008 revision). Written informed consent was obtained from all of the study subjects.

### Study participants and design

All women in the HTDP group met the following criteria: (a) current pregnancy; (b) development of HT before pregnancy, as confirmed by changes in thyroid hormone levels during pregnancy; (c) normal iodine intake; (d) no history of an autoimmune disease other than HT; and (e) no recent history of viral or bacterial infection in the individual or family. HT was diagnosed in accordance with the criteria established by the Endocrinology Society and Perinatal Medicine Branch of the Chinese Medical Association [12] as follows: (a) hardening of the thyroid; (b) abnormal levels of thyroid‐stimulating hormone (TSH), anti‐peroxidase antibody (anti‐TPO), and anti‐thyroglobulin antibody (anti‐Tg); and (c) diagnosis of HT by puncture cytology.

All of the study participants were recruited from Wuhan Children's Hospital (Wuhan, Hubei, China). The study cohort included a total of 13 women with HTDP and 8 healthy controls matched by geography, age, and body mass index.

Whole‐blood samples were obtained from the subjects and collected in tubes containing ethylenediaminetetraacetic acid and stored at −20 °C until DNA extraction.

### Detection of methylation by chip

Genome‐wide methylation analysis was performed using the Illumina Human Methylation 850K Beadchip (Zhuoli Tech, Shanghai, China). Genomic DNA was extracted using the DNeasy^®^ Blood & Tissue Kit (Qiagen, Hilden, Germany) in accordance with the manufacturer's instructions and treated with bisulfite to convert cytosine to uracil. Based on the converted sequences, probes were designed and hybridized with the chip. The ratio of fluorescent signals represents the methylation status.

### DNA methylation quality control and processing

The raw intensity files (idat) were imported into r software (R Foundation for Statistical Computing, https://www.r‐project.org/foundation/) and transformed into β values (range, 0–1) using the ‘minfi’ package for the analysis of Infinium DNA methylation microarray data [13]. Then, quality control was performed by filtering at the sample and probe levels as follows: sites of 1% of the samples with a detection probability (*P*) value of > 0.01 or sites of 5% of the samples with a bead count of < 3 were removed, as were all non‐CpG probes contained in the data set. Multi‐hit probes and probes on the X and Y sex chromosomes were excluded. Additionally, to promote appropriate binding of the probe in the binding area, sites containing single nucleotide polymorphisms were also excluded from the data set. The β values were calculated from the intensity ratio of the methylated signals to the total (methylated and unmethylated) signals for each site, representing the percentage of methylation at a given cytosine residue for all blood cells of an individual. Finally, the PBC algorithm was used to standardize the β value matrix in order to compare the data [14].

### Bioinformatic analysis

Differentially methylated sites (DMSs) between unpaired samples were analyzed using the ChAMP pipeline. Here, a DMP is defined as a single DMS, while a DMR is defined as a cluster of DMSs in the genome. A DMR represents the overall demethylation or hypermethylation status of a particular segment of the chromosome, ranging from hundreds to millions of base pairs.

The DMP function of the ChAMP package and DMSs of the paired samples were analyzed using the equal variance two‐tailed paired *t*‐test. The Benjamini and Hochberg method was performed to control the false‐positive rate when testing multiple hypotheses. For each blood sample, differential methylation was defined as |Δβ| > 0.1 and *P*_adjusted_ < 0.05. For formalin‐fixed paraffin‐embedded samples and cells, the thresholds were set as |Δβ| > 0.2 and *P*_adjusted_ < 0.05. The DMSs were further displayed on each chromosome. The cytobands of the chromosome ideograms were Giemsa‐stained chromosomes obtained from the University of California at Santa Cruz Genome Browser database as defaults.

The gene ontology (GO) of each differentially expressed gene was annotated with reference to the Kyoto Encyclopedia of Genes and Genomes (KEGG) database [15]. GO annotation of the biologic process, cellular component, and molecular function was performed using the DAVID database [16]. A *P* value of < 0.05 was considered statistically significant.

Advanced analysis of the functional epigenetic modules and CNV was also performed. Epigenetic regulation plays an important role in the development, cell differentiation, and etiology of complex diseases, such as cancers. This analysis was conducted using a functional supervision algorithm based on a published protein interaction network. Different modules were grouped based on edge weight assuming that methylation of the promoter region is inversely related to gene expression. A seed gene was defined as a gene with a significant contribution to the observed module. The ‘conumee’ package was used for CNV analysis. The analysis strategy can be summarized in two steps: (a) the adjacent probes were first combined into bins to calculate the degree of variation and (b) the segments were pooled into merged bins.

## Results

### Quality control of genome‐wide detection of methylation with the use of a methylation chip

**Fig. 1:**
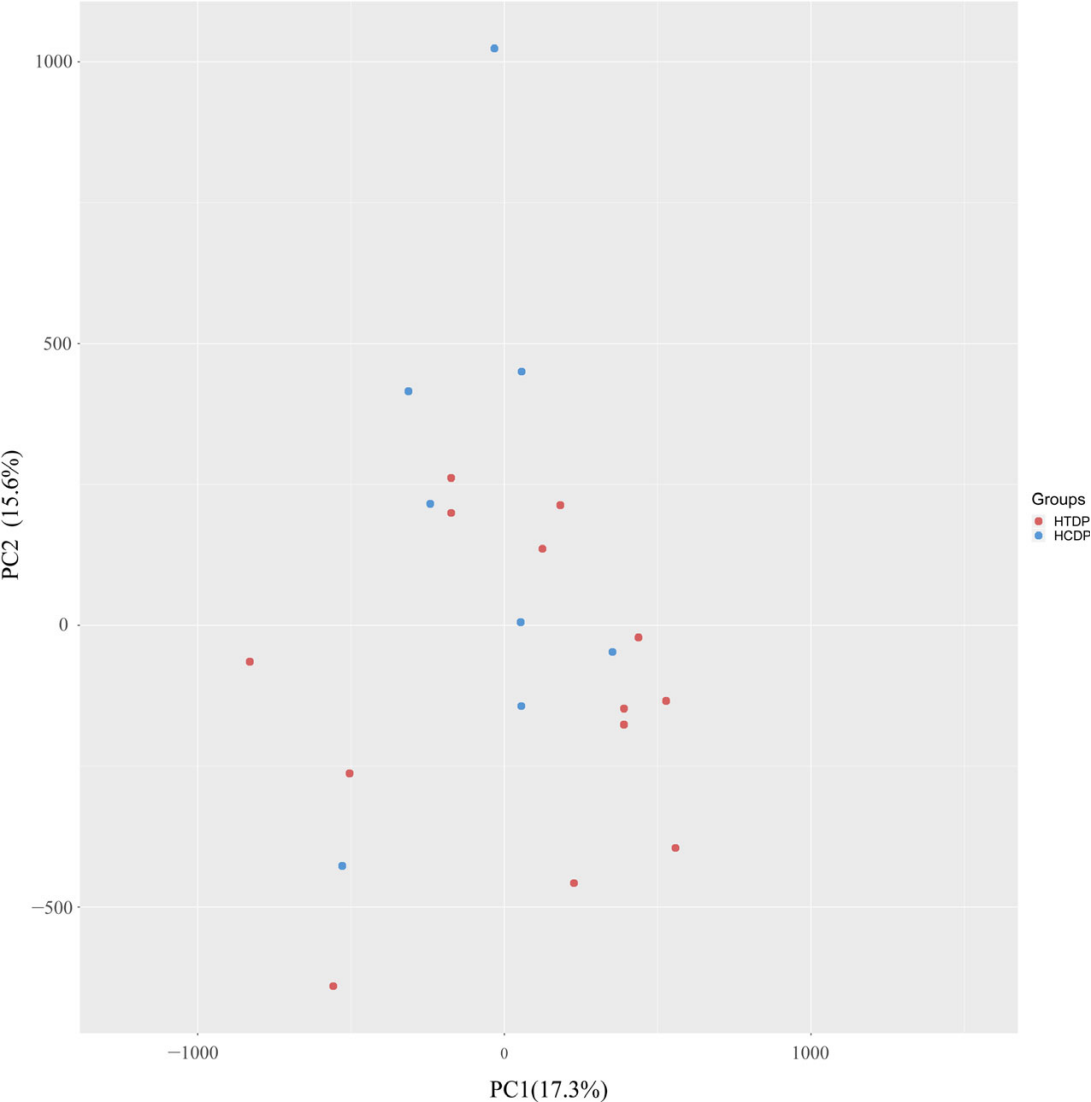
Principal component analysis (PCA) analysis of beta values after standardization of two groups of samples. PC1, principal component 1; PC2, principal component 2.

**Fig. 2:**
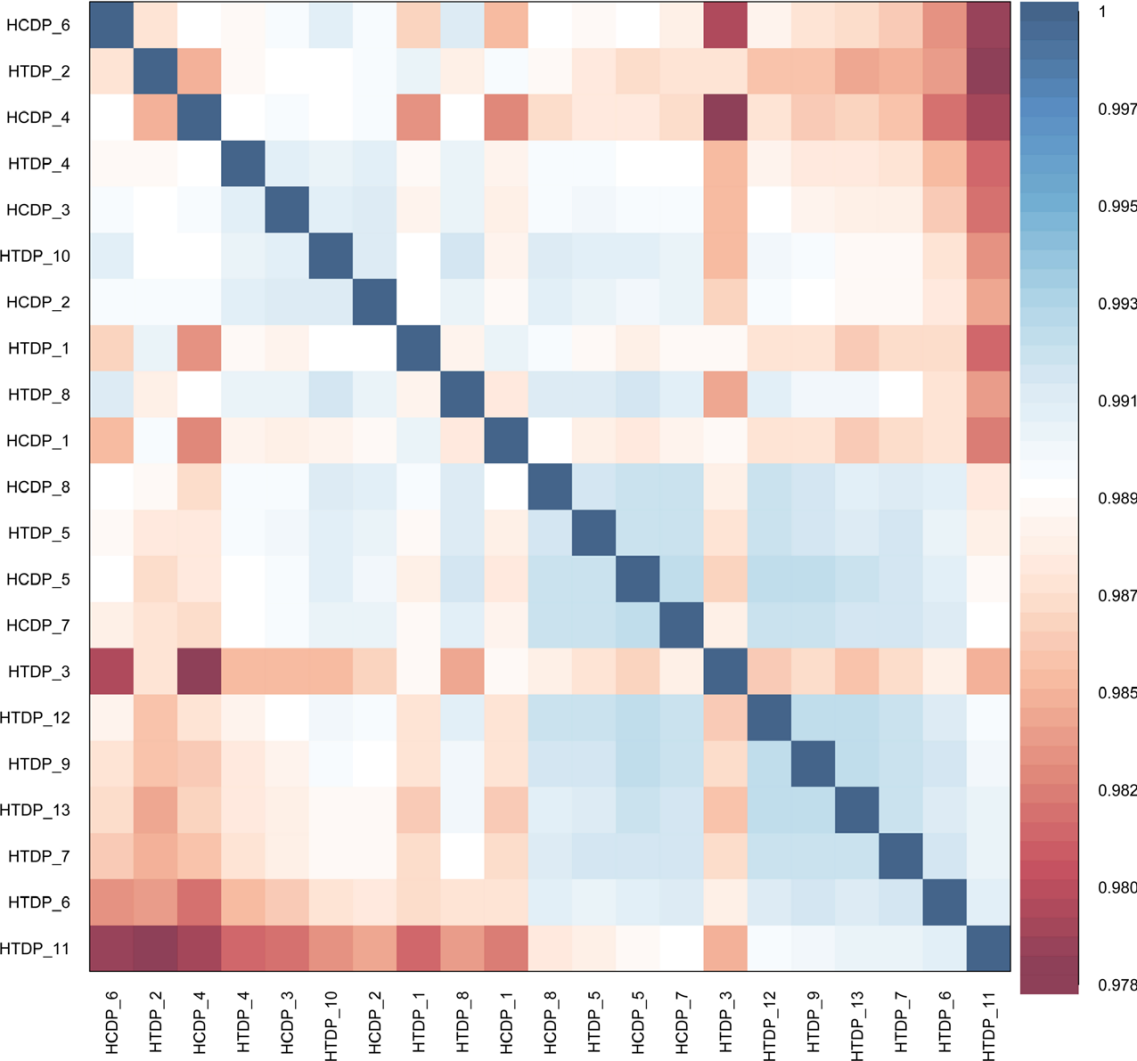
Heatmap of two groups of differentially methylated sites and samples. The color changes from red to blue, indicating that the correlation between the two samples is from low to high.

The data from the HTDP and control samples were preprocessed and normalized. The results of two‐dimensional principal component analysis are shown in Fig. 1. The distance between samples is representative of the expression pattern of an individual gene. Thus, the gene methylation profile of the HTDP group differed from that of the control group. A heat map of the correlations among the samples showed that the gene expression patterns differed among the study participants (Fig. 2).

### Functional analysis of DMPs

**Fig. 3:**
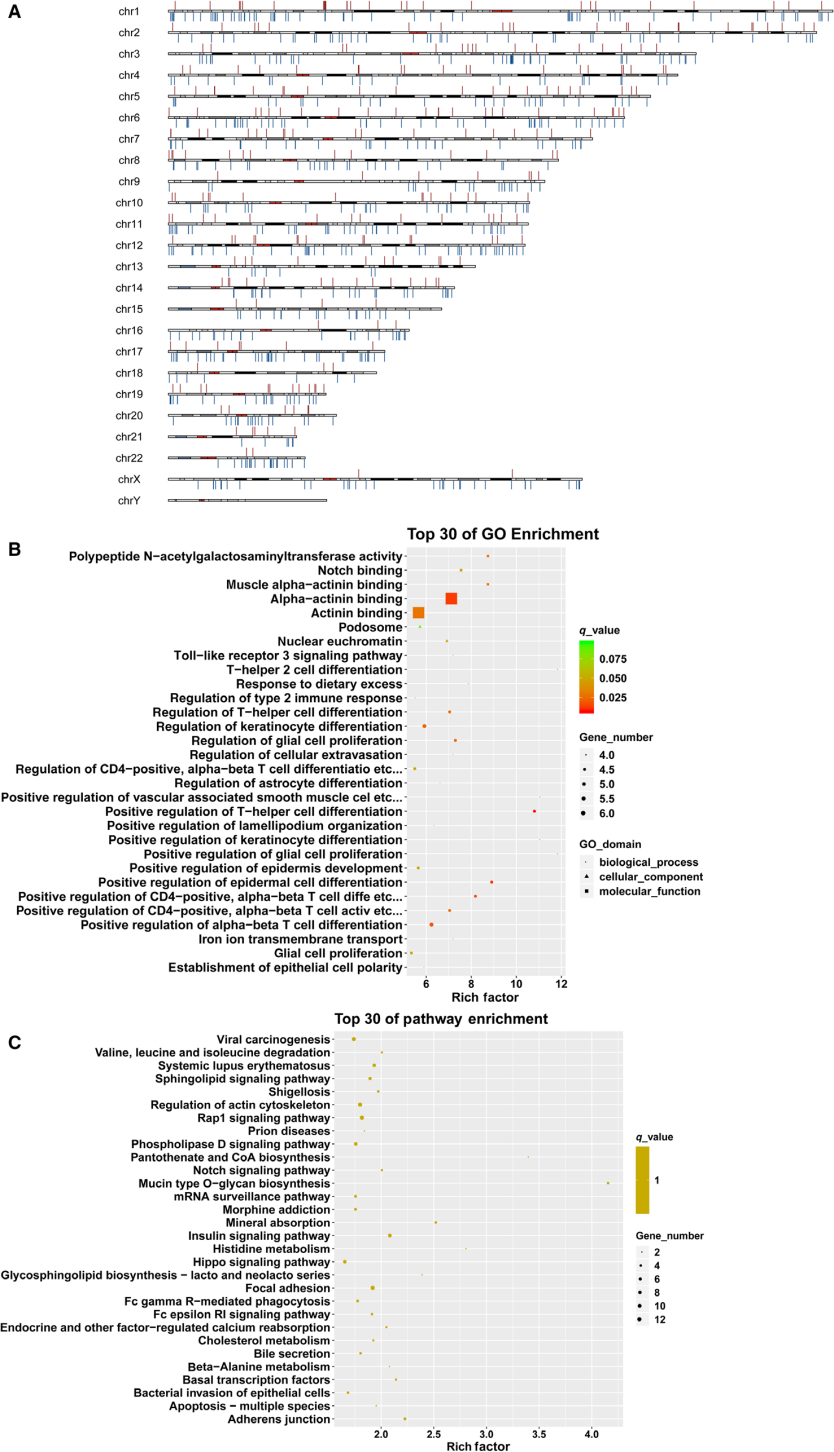
Distribution of methylation sites in the chromosomes and functional analysis of DMPs. (A) The distribution of methylation sites in the chromosomes; (B) the top 30 of gene ontology enrichment; (C) the top 30 of KEGG enrichment.

As compared with the control group, 652 DMPs were identified in the HTDP group. Of the 652 DMPs, 162 were hypermethylated and 490 were hypomethylated. As shown in Fig. 3A, the DMSs of the HTDP group were widely distributed from chromosome 1 to chromosome 22. GO analysis revealed that DMPs were significantly enriched in 540 GO terms. The top 30 GO terms are shown in Fig. 3B. A large proportion of genes with different levels of methylation were significantly enriched in the regulation of keratinocyte differentiation, T helper cell differentiation, and alpha‐beta T‐cell differentiation. Moreover, the significantly enriched KEGG pathways of the DMPs included mucin‐type O‐glycan biosynthesis, focal adhesion, and the insulin signaling pathway (Fig. 3C).

### Functional analysis of DMRs

**Fig. 4:**
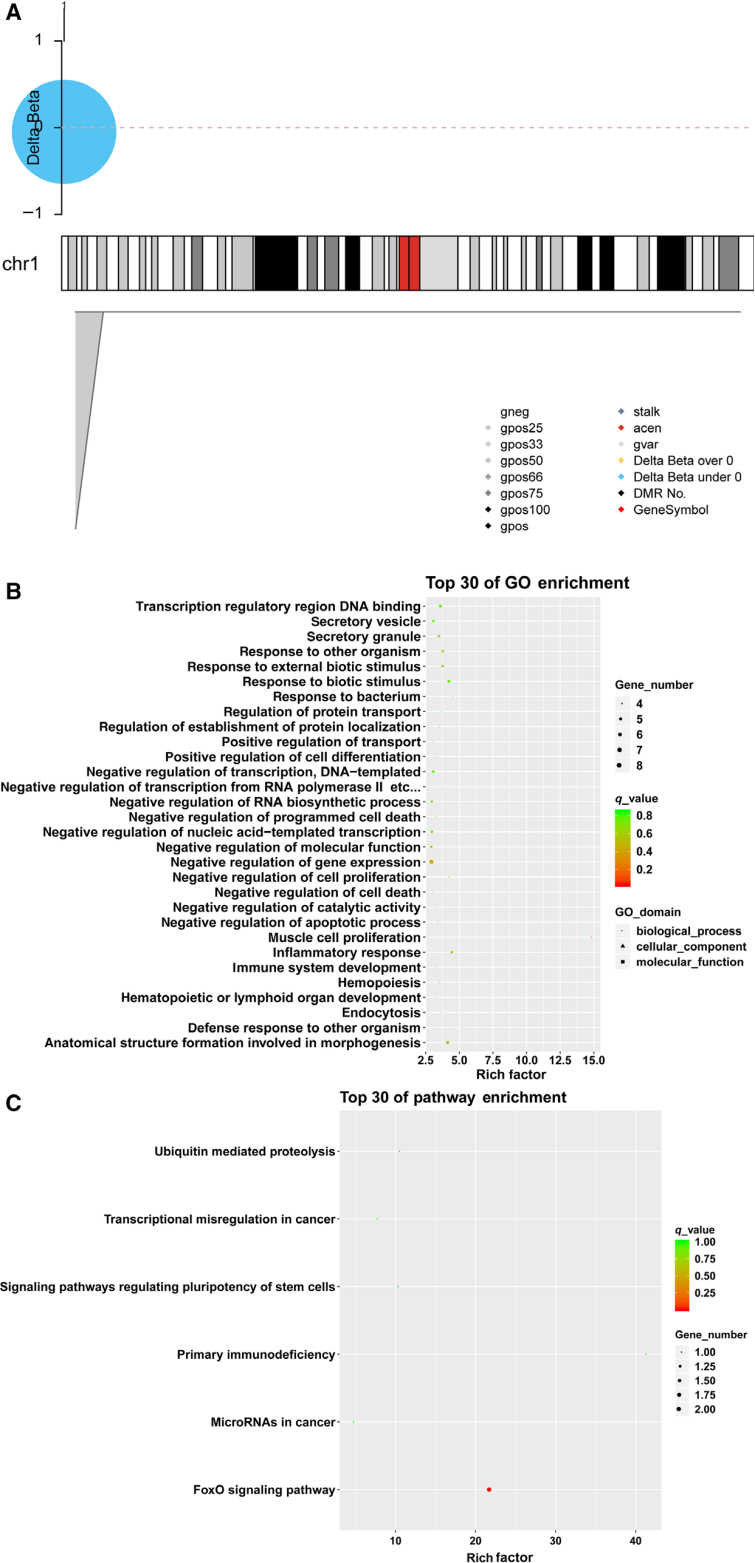
Distribution of methylation sites in the chromosomes and functional analysis of DMRs. (A) The distribution of methylated regions in the chromosomes; (B) the top 30 of gene ontology enrichment; (C) the top 30 of KEGG enrichment.

In total, 27 DMRs were identified in the HTDP group versus the control group, such as MIR140, WWP2, and SLC38A4. Among these 27 DMRs, 15 (55.6%) were distributed along chromosome 1 (Fig. 4A). GO analysis revealed DMRs significantly enriched in 56 GO terms. The top 30 GO terms are shown in Fig. 4B, which including muscle cell proliferation, response to biotic stimulus, and anatomical structure formation involved in morphogenesis. Moreover, the significantly enriched KEGG pathways included the FoxO signaling pathway (Fig. 4C).

### Functional epigenetic modules and CNV

**Fig. 5:**
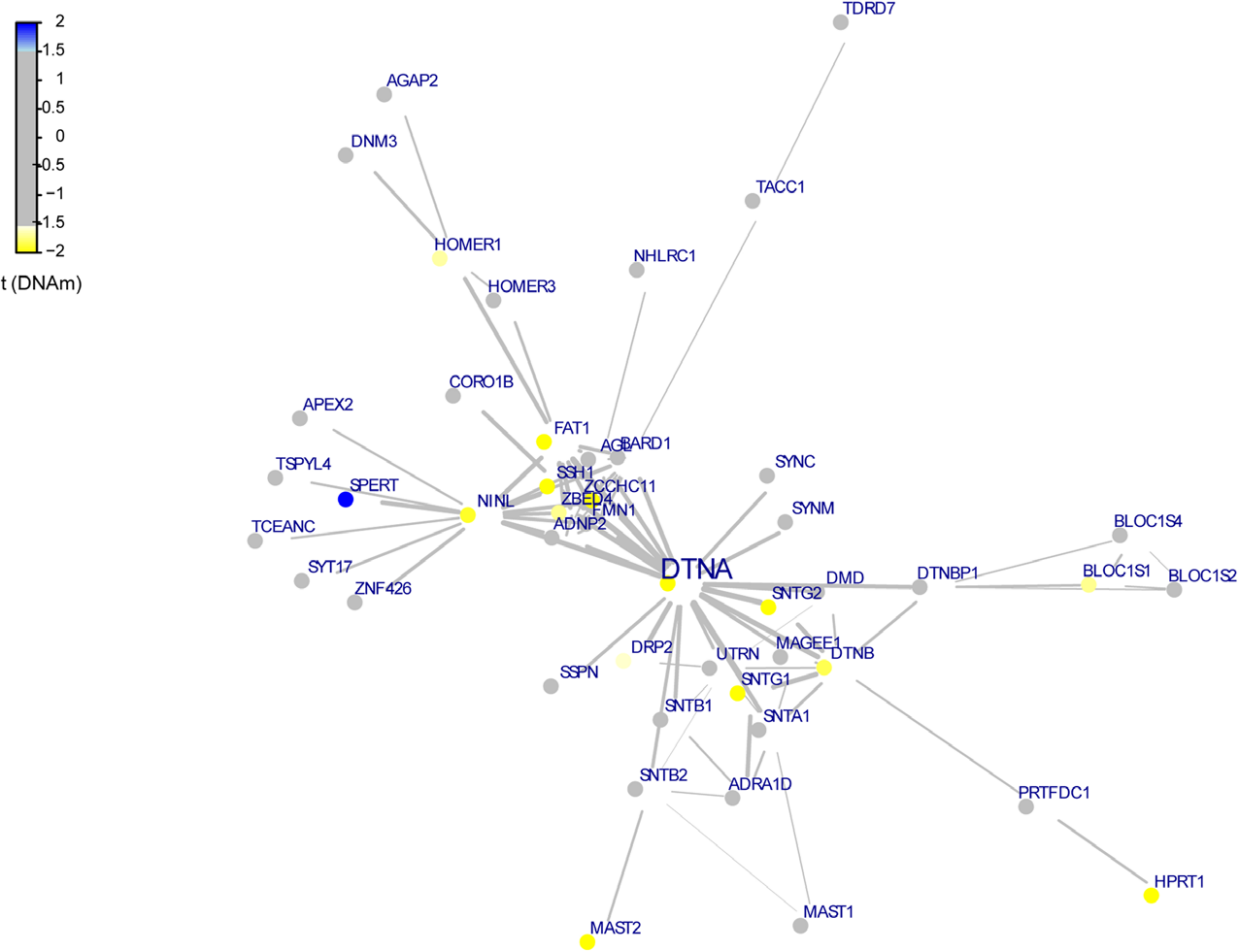
Function epigenetic module display. Each node represents a gene, blue represents positive significance, yellow represents negative significance, and gray represents no difference. The connection between nodes represents the degree of connection between genes, and the thickness of the line represents the level of correlation.

Epigenetic regulation plays an important role in the development, cell differentiation, and etiology of complex diseases, such as cancers. The results of functional epigenetic modules are shown in Fig. 5. In the network, only the SPERT gene played a positive role, while 13 genes, including DTNA, NINL, and ZBED4, played negative roles. Notably, DTNA was the seed gene of the module, suggesting that this gene plays the most important role in the network.

**Fig. 6:**
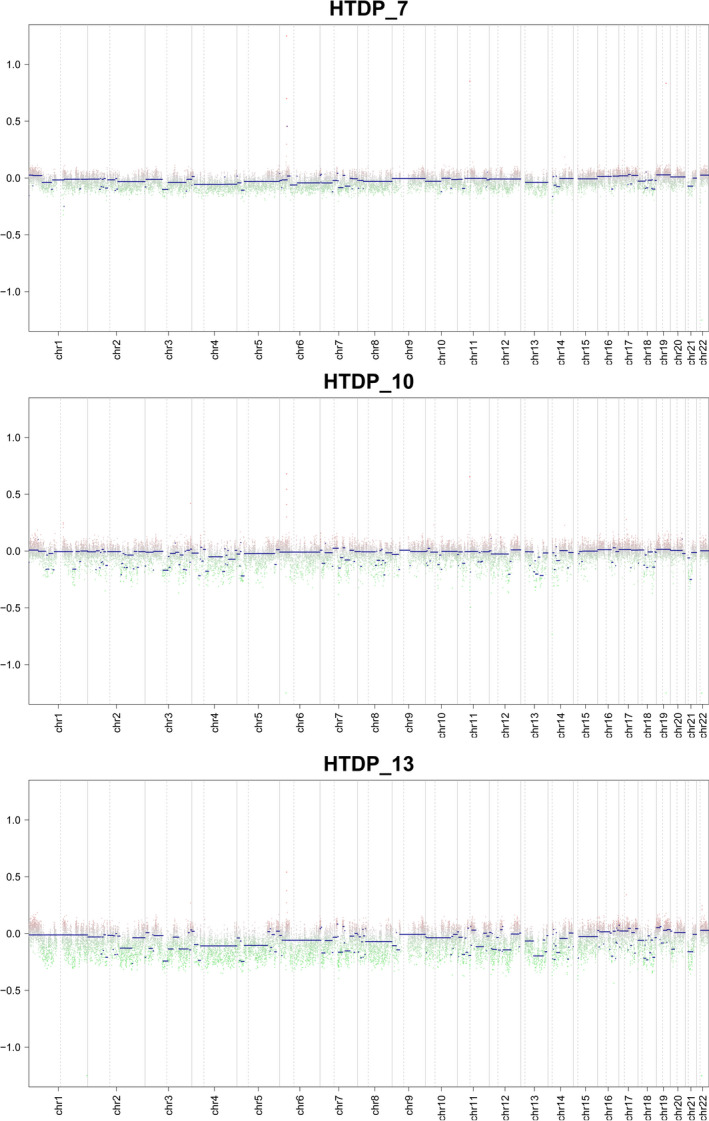
Copy number variation picture of three Hashimoto's thyroiditis during pregnancy samples. The abscissa represents the homochromosomal segment, the ordinate represents the degree of CNV variation, the red and green dots in the figure represent the bins of CNV gain or loss, and the blue line represents the segments.

Copy number variation can influence gene expression levels and several metabolic traits. The results of CNV analysis of 3 (23.1%) of the 13 women in the HTDP group are shown in Fig. 6. Differences in CNV were identified among all women in the HTDP group, suggesting different roles of CNV in the development of HTDP.

## Discussion

In order to explore the DNA methylation status in HTDP, DNA was extracted from 13 women with HTDP and 8 healthy pregnant women as controls. Genome‐wide methylation was detected with the use of an Illumina Human Methylation 850K Beadchip. A total of 652 DMPs and 27 DMRs were identified between the HTDP and control groups. GO analysis revealed DMPs significantly enriched in the regulation of keratinocyte differentiation, T helper cell differentiation, and alpha‐beta T‐cell differentiation. Moreover, the significantly enriched KEGG pathways of the DMPs included mucin‐type O‐glycan biosynthesis, focal adhesion, the insulin signaling pathway, and the FoxO signaling pathway. The significantly enriched GO terms of the DMRs included muscle cell proliferation, response to biotic stimulus, and anatomical structure formation involved in morphogenesis. The DTNA gene was identified as the seed gene of functional epigenetic modules. Our data showed that changes in DNA methylation patterns might influence the development of HT.

Initial clinical signs of autoimmunity diseases associated with thyroid disease are usually related to the skin [17]. The relationship between keratinocytes, as the most common skin cell type, and thyroid disease has been widely reported. For example, Martin *et al*. [18] showed that keratinocyte growth factor contributes to the growth of thyroid cells and Zhang *et al*. [19] proposed that a thyroid hormone analogue could significantly stimulate the proliferation of keratinocytes. A total of six up‐regulated genes were involved in the biological process of keratinocyte differentiation regulation, such as NOTCH1, VDR, and KRT84. Similarly, Molnar *et al*. [20] demonstrated the up‐regulated level of NOTCH1 in HT using a next‐generation sequencing analysis. Moreover, the role of the gene in several autoimmune diseases through modulating T‐cell activation has been reported [21, 22]. In HT, as a kind of TAI, there is a close relationship between the development of the disease and activation pathways of T helper and T regulatory cells [23]. NOTCH1‐dependent may affect the development of HT by regulating T cell‐induced inflammatory. The results of the present study identified DMRs correlated with various biological processes of the GO terms, including muscle cell proliferation, response to biotic stimulus, and anatomical structure formation involved in morphogenesis. Although no clinical data have been published on these correlations, these findings might be useful to further clarify the development of HT.

A previous study reported that protein o‐glycan biosynthesis is related to disease development and plays important roles in protein structure and stability, immunity, and receptor‐mediated signaling [24]. In autoimmune diseases, the expression of adhesion molecules has been associated with leukocyte activation, circulation, and localization. In experimental animal models, the severity of autoimmunity diseases was ameliorated by the administration of antibodies against anti‐adhesion molecules [25, 26]. Although there is currently no evidence of an association between the insulin signaling pathway and TAI, a correlation between TAI and insulin‐dependent diabetes mellitus has been previously demonstrated [27, 28]. The results of the present showed that DMPs were significantly enriched in the FoxO signaling pathway, and three up‐regulated genes were included in the pathway such as SIRT1, MAPK10, and USP7. A previous study demonstrated that FOXO3a was an important factor associated with thyroid carcinogenesis [29]. SIRT1‐mediated abnormal FOXP3 acetylation had a role in regulating regulated T‐cell activation in HT, and regulated T‐cell function deficiency was the main characteristics of HT [30]. Hence, SIRT1 might be a potential treatment biomarker for HT.

In addition, the results of the present study showed that the DTNA gene was the seed gene of the functional epigenetic modules, suggesting an important role of this gene in the development of HT. The DTNA gene encodes α‐dystrobrevin, a member of the dystrophin‐associated glycoprotein complex, which has been associated with movement disorders, such as Duchenne muscular dystrophy [31]. Collins *et al*. [7] demonstrated that the expression level of dystroglycan in the thyroid is regulated by thyroid‐stimulating hormone. Based on the results of the present and previous studies, the role of the DTNA gene in the development of HT should be further investigated.

Both genetic and environmental factors have profound impacts on autoimmune diseases of the thyroid [32]. CNVs are defined as structural variations involving duplications and/or deletions of DNA segments. Traditionally, the development of human diseases is thought to be related to gene loss, duplication, or disruption. In the present study, CNV was identified in all women in the HTDP group. Until now, no data have been reported on genomic CNV in the development of HT. Guan *et al*. [33] demonstrated significant differences in the profiles of the GPC5, B9D2, and ASB11 CNVs in autoimmune diseases of the thyroid. Although the sample size was limited, there were notable differences in the CNVs among all women in the HTDP group. Therefore, further studies are warranted to investigate the association between CNVs and the development of HT.

The present study had several strengths. Illumina 850K Beadchip was used in our study, and the Beadchip covers more DNA methylation sites than the Illumina 27K or 450K. Moreover, the 850K Beadchip double the amount of probes as compared with 450K Beadchip. Thus, it provided us a chance to discover novel role of DNA methylation status in HTDP with more extensive and detailed information on DNA methylation status analysis. Meanwhile, HTDP and healthy controls were matched by geography, age, and body mass index. However, there were still some limitations. Firstly, DNA was extracted from whole blood, which might omit some important methylation loci. Secondly, 13 women with HTDP and 8 healthy pregnant women were involved in the present study, and the limit sample size might restrict the strength of the conclusion. Meanwhile, some baseline data of their subjects such as TSH, anti‐TPO, Tg, and obstetric history could not be obtained, and the influence of these baseline characteristics on the DNA methylation status could not be clarified. Then, further study with more study populations and different characteristics the generalizability would be needed to verify the present conclusion.

## Conclusion

The results of the present showed that the pattern of DNA methylation in the HTDP group was distinct from that of the control group and that DNA methylation may be a mechanism by which genetic polymorphisms contribute to the development of HT.

## Conflict of interest

The authors declare no conflict of interest.

## Author contributions

CW, WF, and XH conceived and designed the project, CW and WF acquired the data; XH and WF analyzed and interpreted the data, CW wrote and revised the manuscript. All authors have read and concurred with the final manuscript.

## Data Availability

Data and analyses will be available from the corresponding author upon reasonable request.
